# Portable 3D-printed electrochemiluminescence sensor for simultaneous glucose and lactate monitoring

**DOI:** 10.1038/s41598-025-18109-4

**Published:** 2025-10-03

**Authors:** Amol S. Kulkarni, Sarika Khandelwal, Kavita Manekar, Purshottam J. Assudani, Rangnath Girhe, Madhusudan B. Kulkarni, Manish Bhaiyya

**Affiliations:** 1 Department of Computer Science and Engineering, G. H. Raisoni College of Engineering, Amravati, 444701 Maharashtra India; 2 Department of Computer Science and Engineering, P. L. Government Institute of Engineering & Technology, Latur, 413512 Maharashtra India; 3 Department of Computer Science and Engineering, G. H. Raisoni College of Engineering, Nagpur, 440016 Maharashtra India; 4 School of Electrical and Electronics Engineering, Ramdeobaba University, Nagpur, 440013 Maharashtra India; 5 School of Computer Science and Engineering, Ramdeobaba University, Nagpur, 440013 Maharashtra India; 6 Department of Electronics and Communication, Government Polytechnic Khamgaon, Khamgaon, 444303 Maharashtra India; 7 Department of Electronics and Communication Engineering, Manipal Institute of Technology, Manipal Academy of Higher Education (MAHE), Manipal, 576104 India; 8 Department of Chemical Engineering and the Russell Berrie Nanotechnology Institute, Technion - Israel Institute of Technology, Haifa, 3200003 Israel

**Keywords:** 3D printing, Biosensor, Electrochemiluminescence, Multi-analyte detection, Interdigitated electrodes, Point-of-care testing, Diagnosis, Biomarkers, Health care, Medical research, Engineering, Nanoscience and technology

## Abstract

Fast and reliable monitoring of metabolic biomarkers, including glucose and lactate, is critical for chronic diseases, sepsis, and other scenarios involving physiological stress. However, most diagnostic options depend on multiple serial tests, require complicated testing apparatus, and are too expensive to be useful in the point-of-care arena. To address these limitations, we present a portable, low-cost, and 3D-printed (3DP) electrochemiluminescence (ECL) biosensor capable of simultaneously detecting glucose and lactate in a single step. The biosensor incorporates interdigitated electrodes (IDEs) within a bipolar electrode (BPE) electrochemical system to enhance electron transfer and signal amplification. Through careful optimization of voltage, luminol concentration, and pH, strong and reproducible ECL signals were achieved, with detection limits of 0.1 mM for glucose (linear range 0.1 mM to 5.0 mM) and 80 µM for lactate (linear range 0.1 mM to 4.0 mM). Real serum analysis yielded 95–102% recovery rates, validating the device’s clinical relevance. Combining an inexpensive smartphone-based readout and fabricated with low-cost carbon and polylactic acid (PLA) filaments, this device offers a scalable and field-deployable approach to real-time, decentralized diagnostics. The proposed prototype fills a critical void between laboratory analysis and standard healthcare needs and promotes accessible and affordable metabolic monitoring for underserved populations.

## Introduction

As we move towards personalized medicine and decentralized diagnostics, the need for platforms that can quickly and accurately detect biomarkers from human samples is rapidly increasing^1,2^. From the management of chronic diseases like diabetes to reaction to acute scenarios like sepsis or trauma, clinicians are increasingly relying on real-time biochemical markers (biomarkers) in their decision-making processes. Among these, glucose and lactate are two critical biomarkers, routinely monitored to assess metabolic status, detect physiological stress, and diagnose systemic complications^3–5^. Notably, traditional means of measuring important analytes, including colorimetric assays, benchtop electrochemical analyzers, and optical biosensors, often fail to conform to the rigors of the Point of Care (POC) settings in terms of their complexity, cost, and necessity for sequential measurement^6–8^.

The crux of the problem lies in the instrumentation and the inability of most conventional systems to detect multiple analytes in a single step simultaneously. Most systems rely on dedicated, analyte-specific setups that are time-consuming and impractical for real-world clinical workflows^9,10^. Multiplexing, detecting more than one biomarker at once, has been a longstanding challenge due to issues like signal interference, cross-reactivity, and complex assay configurations. These issues limit both the throughput and accuracy of diagnosis, especially in high-stakes scenarios like emergency care or remote health monitoring^11–13^.

Biosensor technology has seen significant innovation in recent years to address these shortcomings. Among the various platforms explored, electrochemiluminescence (ECL) has emerged as a powerful biosensing mechanism due to its high sensitivity, low background noise, and compatibility with miniaturized formats^14–16^. Unlike fluorescence-based systems that require external light excitation (and hence introduce optical noise), ECL signals are generated electrochemically, ensuring improved signal-to-noise ratios. This makes ECL systems highly appealing for portable, field-deployable diagnostics. However, despite these advantages, traditional ECL sensors typically focus on single-analyte detection. This limits their utility in conditions where multiple biomarkers must be monitored together to provide clinically relevant insights^17–19^.

Moreover, achieving reliable multi-analyte detection using ECL faces several bottlenecks. Firstly, the surface area available on traditional planar electrodes is often insufficient for supporting multiple spatially isolated reactions^20,21^. Secondly, fabrication methods such as screen printing or photolithography, while precise, are expensive, labor-intensive, and not easily scalable for on-demand sensor production. Lastly, the lack of affordable and portable ECL detection setups continues to limit their widespread adoption beyond research labs^22,23^.

To overcome these issues, researchers have explored the integration of interdigitated electrodes (IDEs) into ECL biosensors^24,25^. IDEs offer a substantial advantage by increasing the electroactive surface area and enabling redox cycling, which improves signal amplification^22,26^. Their unique geometry also supports spatial segregation of reactions, reducing signal cross-talk and enabling multiple analytes to be monitored in parallel. However, the cost and complexity of manufacturing IDE-based sensors using conventional techniques have hindered their accessibility^27,28^.

This is where 3D printing (3DP) enters the narrative as a transformative enabler. With the capability to rapidly fabricate complex geometries using conductive and non-conductive filaments, 3DP offers a cost-effective, scalable, and design-flexible alternative to traditional electrode fabrication. By combining 3DP with IDE-based ECL sensing, it becomes possible to create customizable, miniaturized, and low-cost multi-analyte biosensors that are suitable for real-world diagnostics^29–31^. Recent advancements in 3DP technologies have enabled precise deposition of carbon-loaded polylactic acid (PLA) filaments to construct the conductive regions of biosensors, while structural components can be simultaneously printed using standard PLA. This dual-material printing allows seamless fabrication of devices with integrated electrodes and fluidic architecture, eliminating manual assembly and reducing fabrication time. Furthermore, the use of smartphone-based imaging systems in place of bulky CCD detectors or PMTs enhances portability, making the system suitable for field use. While earlier efforts have showcased the use of 3DP-ECL sensors for individual analyte detection, simultaneous detection of multiple biomarkers, especially using fully integrated IDEs in a compact, smartphone-readable format, remains underexplored. Although theoretically feasible, multiplexed ECL sensing is often limited by signal overlap, reaction zone interference, and non-scalable fabrication approaches^32,33^.

To address these pressing challenges, we introduce a novel 3DP-based ECL biosensor integrating interdigitated electrodes within a bipolar electrochemical system to detect glucose and lactate simultaneously. The innovation lies in combining 3DP and IDEs and leveraging spatially separated reaction wells, redox cycling for signal amplification, and smartphone-based detection, all in a compact, cost-effective platform. The use of additive manufacturing further facilitates on-demand production, adaptability to other biomarkers, and deployment in low-resource environments. This work bridges a critical gap between high-performance ECL biosensing and real-world applicability. By providing interference-free, low-cost, and scalable simultaneous multi-analyte detection, our proposed biosensor represents a significant step forward in real-time metabolic monitoring, particularly for decentralized and point-of-care diagnostics.

## Experimental section

### Materials and equipment used

This study involved the use of various chemicals and biomolecules, including luminol, glucose, cholesterol, lactate, lactate oxidase (LOx), choline, glucose oxidase (GOx), creatinine, vitamin B12, bovine serum albumin (BSA), and choline, all sourced from Sigma Aldrich, India. Since luminol dissolves better in a basic solution, a sodium hydroxide (NaOH) solution was first prepared using NaOH procured from Sisco Research Laboratories, India. The luminol stock solution (10 mM) was prepared following a previously published method from the research group. This involved dissolving luminol in 50 mL of deionized (DI) water, which consisted of 47 mL of DI water and 3 mL of NaOH (0.1 M concentration) to maintain the required basic conditions. Using this stock solution, different luminol concentrations (1 mM to 7 mM) were prepared using a standard dilution technique in DI water. Similarly, 10 mM glucose and choline stock solutions were prepared in DI water and diluted accordingly^19^. Additional solvents, including isopropanol (IPA) and dimethylformamide (DMF), were procured from SRL, India. The recording of ECL signals was comprehensible and consistent by simply keeping a Motorola G45 (50 MP camera) at an optimal distance above a 3D-printed black box, which facilitated signal recording behaviour. To power this ECL device without using an external power supply, a DC-DC buck-boost converter was used, which was variable from 2.4 V to 24 V. The added design choice improved the portability and operational efficiency of the system, making it easier to use in electrochemiluminescence-based sensing applications.

The 3DP ECL biosensor was fabricated using a FlashForge Creator 3 Pro dual-extrusion FDM 3D printer, which provides high dimensional accuracy and multi-material compatibility. Two types of filaments were employed: a conductive carbon-loaded PLA filament (1.75 mm, Protopasta, USA) for printing the IDE zones, and a standard white PLA filament (1.75 mm, Amazon India) for structural housing and support. The printer was configured with a 0.4 mm nozzle, a layer height of 0.2 mm, and a printing speed of 40 mm/s to ensure precise extrusion and definition of the narrow IDE fingers. The nozzle temperature was set to 220 °C, with the heated bed maintained at 60 °C to promote adhesion and layer integrity. The infill density was set to 100% using a rectilinear infill pattern to ensure high electrical conductivity in the electrode regions. This approach allowed us to print both conductive carbon-loaded PLA and non-conductive white PLA in a single step (simultaneously), eliminating the need for manual assembly or adhesive bonding. The 3D design was prepared in CAD s(Full Name: Autodesk Fusion360; Version: v.2.0.19941; URL Link: https://www.autodesk.com/in/) with precise zoning to assign the conductive material exclusively to the electrode regions (interdigitated fingers and contact pads), while the structural components, such as the sensor body and fluidic wells, were assigned to the white PLA extruder. The slicing software -FlashPrint (Full Name: FlashForge FlashPrint; Version: 5.5.0; URL Link: https://www.flashforge.com/) enforced proper alignment and layer-by-layer fusion between the two materials. The overlapping design margins and tight tolerances in the CAD model further ensured interlocking of layers, minimizing the risk of fluid leakage across the interface.

**Fig. 1:**
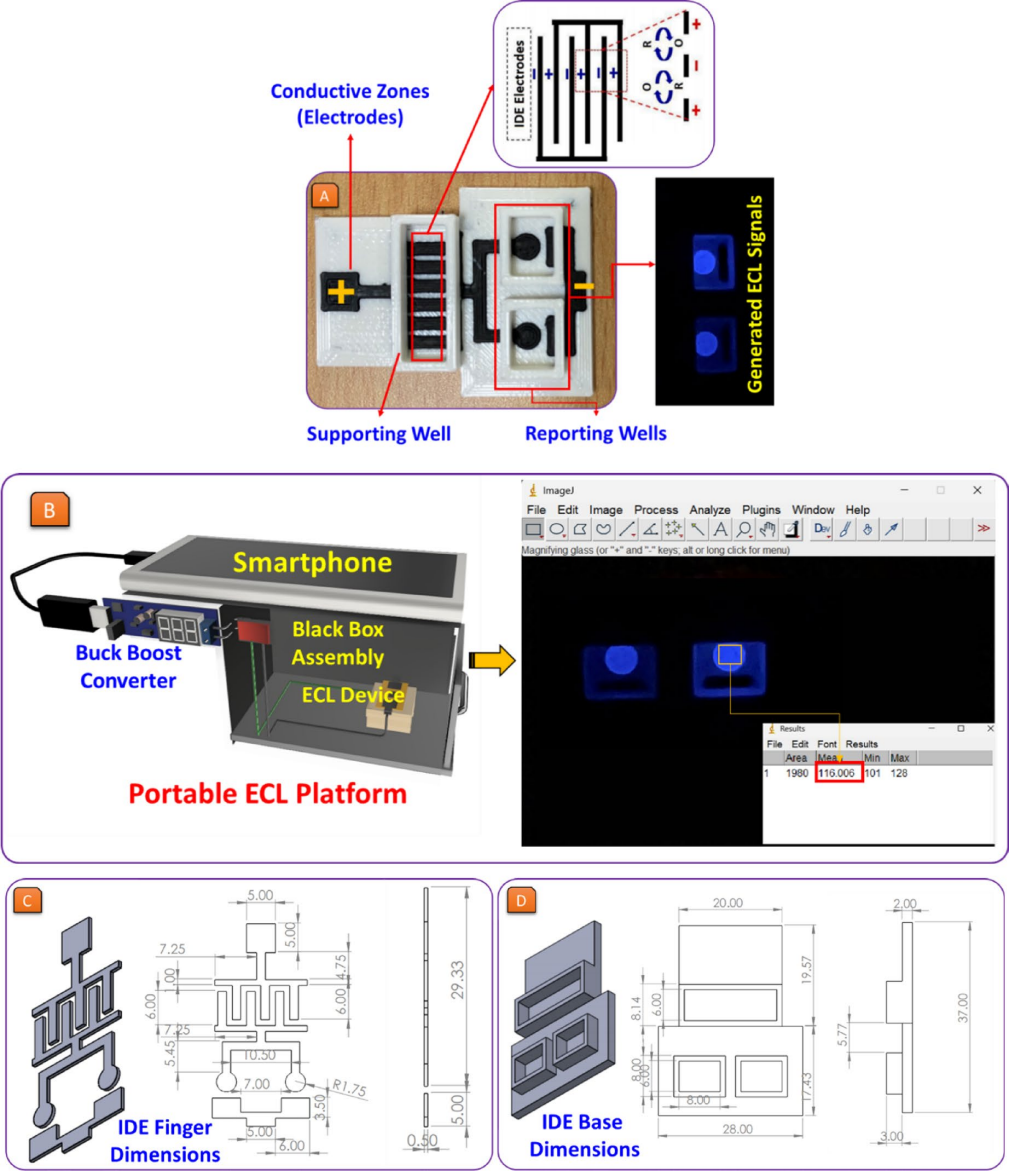
(**A**) Schematic and real image of the 3DP ECL biosensor, highlighting key components: conductive IDE zones, supporting well, and reporting wells for multi-analyte detection. (**B**) The portable ECL detection platform, consisting of a black box assembly, buck-boost converter, and smartphone-based imaging system, is designed for real-time and point-of-care biomarker detection. (**C**) CAD design and dimensional specifications of the IDE layout used in the 3D-printed biosensor. (**D**) Structural dimensions of the 3DP base supporting the IDE configuration.

Support structures were enabled for overhangs in structural components but disabled around the electrode zones to avoid surface obstruction. The printed biosensor was oriented horizontally during fabrication to maintain the dimensional integrity of the 0.5 mm-wide IDE fingers and 0.5 mm inter-finger gaps. Post-printing, the electrode regions were gently polished using fine-grit sandpaper to remove surface irregularities and enhance electrical contact. No additional chemical or thermal post-processing was applied. The IDE geometry, which includes six pairs of interdigitated fingers (0.5 mm width, 0.5 mm spacing), was designed to facilitate redox cycling and improve ECL signal amplification. Figure 1 (C–D) provides a CAD-based schematic with full-dimensional annotations. These fabrication parameters ensure reproducibility, low cost, and mechanical and electrical robustness, supporting the biosensor’s application in point-of-care diagnostics. Following printing, the device was visually inspected, rinsed with isopropanol, and tested with buffer solutions to confirm the leak-tightness of the well chambers. No delamination or leakage was observed, validating the robustness of the single-step dual-material printing strategy. This fully integrated fabrication process significantly simplifies device production, improves reproducibility, and makes the biosensor platform suitable for scalable deployment in resource-limited settings.

### Fabrication of ECL device

A 3DP approach was used to fabricate the ECL device for precise, reproducible, and customizable manufacturing. First, using SolidWorks software (Full Name: Dassault Systèmes SolidWorks Corporation; Version number: SolidWorks 2024; URL Link: https://www.solidworks.com/), the device was modeled using electrode arrangements and fluidic wells over CAD. After finalizing the design, the model was sent to the 3D printer through its graphical user interface (GUI) for printing execution. While fabricating the device, the bed temperature was maintained at 60 °C, and the PLA filament was heated to 220 °C to obtain suitable printing quality and printing structure. The functional ECL biosensors were successfully fabricated using real-time extrusion and optimized layer adhesion parameters. The working principle of the ECL biosensor involves emission of light during electron transfer reactions. The detection mechanism relies on electrochemically generated reactive species, which undergo oxidation-reduction (redox) reactions to produce a luminescent signal. The intensity of this signal will correspond to the concentration of the target analyte, allowing quantitative detection of diverse biomarkers. ECL biosensors find widespread applications because of their high sensitivity, low background signal, and selective detection abilities in medical diagnostics, environmental monitoring, and biochemical analysis.

The IDEs incorporated into the structure of this biosensor significantly improve the surface area for oxidation and reduction reactions. The larger surface area makes the electrons transfer more effectively, strengthening the signal and enhancing detection. These IDEs are integrated into a BPE configuration, where a single pair of electrical contacts can operate multiple IDE-BPE units. The BPE system consists of a cathode and an anode, with the cathode being divided into various interdigitated arms. The arms merge with the anode in this way so as not to hinder redox activity. When a sufficient potential is applied, an oxidation reaction at the anode and a reduction reaction at the cathode generate the ECL signal at the anode. A light is then emitted, which is detected and analyzed for the route to biomolecule detection^25,34^. Figure 1 (C) illustrates that the IDE comprises six interdigitated finger pairs, each with a finger width of 0.5 mm and inter-finger spacing of 0.5 mm. Figure 1 (D) shows that the base includes two isolated reporting wells (each 8 mm × 8 mm) and a central supporting chamber, maintaining physical separation to prevent cross-talk between analytes. These CAD-optimized design features were critical for achieving high sensitivity, reproducibility, and independent detection of glucose and lactate in a miniaturized and cost-effective format.

The 3DP design of this biosensor has several advantages over traditional designs. Firstly, there is greater customization and flexibility due to the ability to modify the design using CAD software. The design can amplify the geometric features of the electrode and dimensions of the well. Further, 3DP greatly reduces the manufacturing cost when compared to conventional photolithography/electrode deposition techniques. The device is compact, lightweight, and uses a simple power source (DC-DC buck-boost converter), making it suitable for field deployment. The conductive carbon filament used to fabricate electrodes has excellent conductivity and durability, which helps enhance the performance of sensors^35–37^. Figure 1(A) depicts a schematic of two well-3DP ECL biosensors showing their components and working mechanism. The combination of this method of fabrication with the high sensitivity of ECL-based detection ensures that the system is effective in biomedical diagnostics, environmental monitoring, and biochemical research.

### Enzyme functionalization of reporting wells

Following fabrication, each reporting well of the 3DP biosensor was functionalized independently with its respective enzyme for selective detection. 10 µL of glucose oxidase (GOx, 10 mg/mL in PBS, pH 7.4) was carefully pipetted into the designated glucose well for glucose detection. For lactate detection, 10 µL of lactate oxidase (LOx, 50 U/mL in PBS) was similarly added to the lactate well. The biosensor was then incubated at room temperature in a humid chamber for 30 min to facilitate physical adsorption of the enzyme onto the carbon electrode surface. After incubation, the wells were gently rinsed with PBS to remove any loosely bound enzyme and subsequently treated with 1% bovine serum albumin (BSA) for 10 min to block non-specific binding. A final PBS rinse was performed, and the fully functionalized biosensor was stored at 4 °C until further use.

Before each experiment, the biosensor was retrieved from refrigeration and allowed to equilibrate to room temperature for a few minutes. Then, 30 µL of the target analyte (glucose or lactate) and 30 µL of luminol (4 mM) were pipetted into the respective reporting well. The mixture was incubated for 90–120 s to allow the enzymatic reaction to occur, during which the action of GOx or LOx generated H₂O₂. This H₂O₂ then reacted with luminol under applied voltage conditions to initiate the ECL reaction, producing a luminescent signal proportional to the analyte concentration. Importantly, the supporting well contained only the electrolyte, which served as the ionic bridge in the bipolar electrochemical system. All wells operated independently without observable cross-talk, enabling reliable and simultaneous multi-analyte detection.

### Detection principle and ECL mechanism

The ECL biosensor follows the bipolar electrochemical principles established in a previous study^25,34^, and this design is essential for signal amplification so the biosensor can detect biomarkers with high sensitivity.

The fundamentals of the biosensor design include conductive electrode zones, supporting and reporting wells, and a device or mobile operating system to perform the detection. IDEs allow electrochemical reactions to happen simultaneously by redox cycling, which transfers electrons between the two closely spaced electrodes to increase the ECL signal for detection. The biosensor operates by coupling enzyme-generated H_₂_O_₂_ to the classic luminol ECL pathway within electrically isolated, reagent-specific wells. Each reporting well is functionalized with an enzyme substrate pair, glucose/GOx or lactate/LOx, so that, after the sample is introduced, the respective oxidase converts dissolved O₂ to H₂O₂ proportionally to analyte concentration. A brief incubation ensures sufficient H₂O₂ accumulation. When a potential is then applied across the feeder electrodes, the interdigitated microbands inside each well act as an IDE-BPE, creating closely spaced anodic and cathodic regions that sustain intensive redox cycling. At the anodic fingers, luminol is electro-oxidized to the diazoquinone intermediate (Eq. 1), which reacts chemically with the locally produced H₂O₂ to form the luminol endoperoxide (Eq. 2). This endoperoxide decomposes to nitrogen and the electronically excited 3-aminophthalate (Eq. 3), which relaxes radiatively to its ground state with photon emission (hν) that constitutes the ECL signal (Eq. 4). The diffusion paths between adjacent IDE fingers continuously regenerate reactive species, so oxidation and reduction proceed repeatedly at high efficiency, markedly amplifying light output. Because each well operates independently, glucose and lactate are quantified simultaneously without cross-interference, and the smartphone camera records the emitted intensity from each well; after calibration, that intensity is directly proportional to analyte concentration. The combination of IDE-BPE geometry, enzyme-driven H₂O₂ production, and luminol ECL thus delivers a robust, sensitive, and scalable platform suitable for biomedical diagnostics, metabolic monitoring, and clinical research. The following are the most widely used ECL reactions^38,39^.

1 $${\text{Luminol}}\, - \,{{\text{e}}^ - } - {{\text{H}}^ - } \to {\text{Diazoquinone}}$$

2 $${\text{H}}_{{\text{2}}} {\text{O}}_{2} + {\text{ Diazoquinone}} \to {\text{Luminolendoperoxide}}$$

3 $${\text{Luminolendoperoxide}} \to {\text{N}}_{2} + {\text{ 3}} - {\text{aminophthalate}}*$$

4 $${\text{3}} - {\text{aminophthalate}}* \to {\text{3}} - {\text{aminophthalate}}\,+\,{\text{h}}$$

### Data acquisition and analysis

In order to precisely capture and quantify the ECL signal, a closed black box assembly was constructed using 3DP to prevent any ambient light from affecting the measurements. A DC-DC buck-boost converter, which was embedded into the enclosure and powered the circuit, converts the 5 V output from a smartphone USB port to a tunable voltage (3–23 V). This setup, previously reported by the research group^40,41^, ensured a reliable and portable ECL detection system. The optimized voltage was then applied to the interdigitated electrodes of the ECL biosensor to initiate the ECL reaction. A Motorola G45 smartphone equipped with a 50 MP camera was positioned 10 mm above the detection zone for signal acquisition. The camera recorded 30 frames per second, and the frame with the highest luminescence was manually selected for analysis. The selected image was subsequently analyzed using the open-source image analysis software ImageJ (Full Name: An open-source image processing and analysis software; Version: 1.54p 17- ImageJ2; URL Link: https://imagej.net/ij/) to measure the light intensity. The generated ECL signal is expressed as Relative Light Units (RLU), a conventional unit representing the total brightness level emitted in arbitrary units during the ECL reaction. This approach enabled cost-effective, portable, and reliable quantification of ECL signals without the need for photomultiplier tubes or CCD cameras^16,42,43^. This arrangement, consisting of a smartphone camera, a stable power supply, and effective image analysis software, makes it a convenient and efficient platform for making ECL measurements in real-time or on-the-go. Figure 1 (B) shows a diagram of the ECL imaging system integrated with the smartphone, highlighting how this simple, unobtrusive approach enables accurate detection and biosensing applications of biomarkers.

## Results and discussion

### Parameter optimization

Optimizing the key parameters of an ECL biosensor is essential for achieving high sensitivity, stability, and reliable detection of target analytes. Without proper optimization, the system may suffer from low signal intensity, background noise, inefficient electron transfer, or unnecessary side reactions, all of which can impact accuracy. Since ECL is driven by electrochemical reactions, factors like applied voltage, luminol concentration, and pH must be carefully fine-tuned for the best possible performance. The goal is to ensure the biosensor produces a strong, stable, reproducible luminescence signal under optimal conditions.

**Fig. 2:**
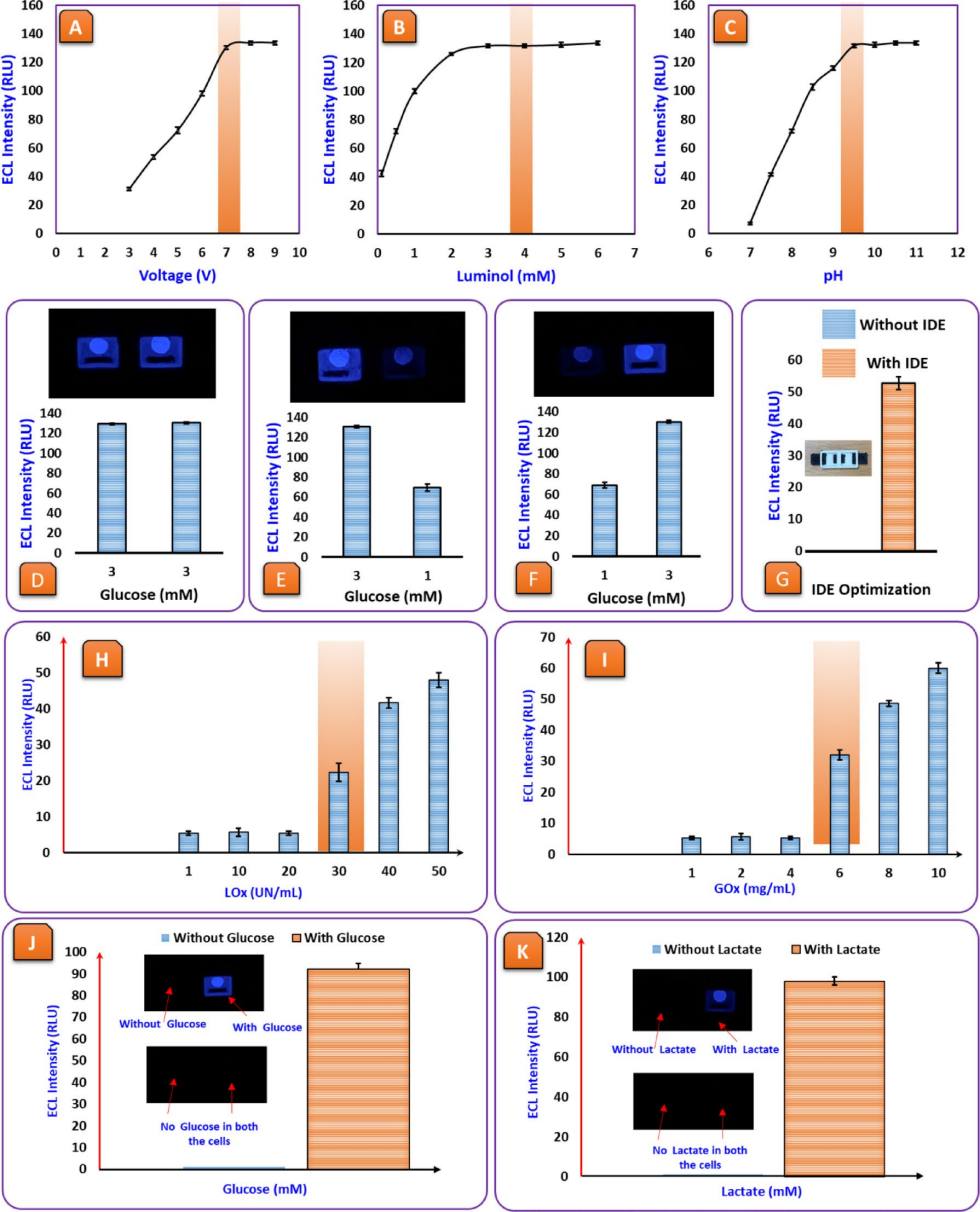
(**A**) Effect of applied voltage on ECL response: voltage (3 V to 10 V), Luminol (5 mM), pH (10), Glucose (1 mM), GOx (10 mg/mL). (**B**) Effect of luminol concentration: voltage (7 V), Luminol (0.1 mM to 6 mM), pH (10), Glucose (1 mM), GOx (10 mg/mL). (**C**) Effect of pH: voltage (7 V), Luminol (4 mM), pH (7 to 11), Glucose (1 mM), GOx (10 mg/mL). (**D**–**F**) Validation of independent well detection for simultaneous glucose measurement: (**D**) Identical glucose concentrations (3 mM) in both wells resulted in equal ECL signals, confirming uniform response, (**E**) Different glucose concentrations (3 mM and 1 mM) in separate wells showed proportional ECL intensities, validating concentration-dependent response, (**F**) Swapping the glucose concentrations between wells maintained the expected intensity pattern, confirming independent well functionality without cross-interference. (**G**) IDE Optimization: voltage (7 V), Luminol (4 mM), pH (10), Glucose (0.5 mM), GOx (10 mg/mL). C) Effect of pH: voltage (7 V), Luminol (4 mM), pH (7 to 11), Glucose (1 mM), GOx (10 mg/mL). (H) Effect of varying LOx concentrations (1–70 U/mL, Luminol = 4 mM, Voltage = 7 V, Lactate = 1 mM) on the ECL intensity, maximum ECL signal is observed at 50 UN/mL, beyond which the signal stabilizes. (I) Effect of varying GOx concentrations (1–14 mg/mL, Luminol = 4 mM, Voltage = 7 V, Glucose = 1 mM) on ECL intensity. Maximum ECL signal is observed at 10 mg/mL, beyond which the signal stabilizes, confirming optimal enzyme loading for biosensor performance. (J) Background signal and Specificity testing: voltage (7 V), Luminol (4 mM), pH (10), Glucose (2 mM), Lactate (2 mM), GOx (10 mg/mL), LOx (30 UN/mL). Error bars represent standard deviations (*n* = 3).

The first experiment focused on voltage optimization (Fig. 2. (A)) by varying the applied voltage from 3 V to 10 V, while keeping all other parameters constant. The results showed a steady increase in ECL intensity as the voltage increased, followed by a saturation point where the signal stopped rising. The electron transfer process is weak at lower voltages (3–5 V), leading to a dim ECL signal. As voltage increases, more electrons drive the redox reactions, causing the signal to grow stronger. However, beyond a certain voltage, the system reaches its maximum efficiency, meaning no further voltage increase will improve the signal. This happens because the reactive species responsible for the luminescence are already being produced at their highest possible rate. Applying too high a voltage can even lead to side reactions, excessive background noise, or degradation of key components, making it essential to identify the ideal voltage for a stable and strong signal^44–46^.

Next, luminol concentration optimization (Fig. 2 (B)) was performed by varying the amount of luminol, the primary ECL luminophore, to study its effect on signal intensity. The data showed that increasing the luminol concentration initially resulted in a stronger ECL signal, but after a certain point, the signal plateaued or slightly decreased. Fewer molecules participate in the reaction at low luminol concentrations, producing a weak signal. The ECL reaction intensifies as more luminol becomes available, leading to brighter luminescence. However, beyond the optimal level, adding more luminol does not help; it can cause self-quenching, where excessive molecules interfere with each other, reducing efficiency. This means that finding the right luminol concentration is critical to avoid both low signal output and unnecessary chemical interference^47,48^. The final parameter tested was pH optimization (Fig. 2. (C)), which plays a crucial role in stabilizing the reaction environment and controlling the efficiency of luminol oxidation. The results showed that the ECL signal increased as pH was adjusted to an optimal range, but beyond that, the signal stabilized or even dropped. This is because the reaction kinetics are sluggish at low pH levels, leading to weak ECL emission. As pH rises, the electrochemical reaction speeds up, boosting luminescence. However, if the pH becomes too high, luminol degradation or side reactions can occur, negatively affecting signal intensity. This confirms that maintaining a balanced pH range is essential to ensure the best possible performance of the biosensor^47,48^.

### Dependency check

The experiment aims to check whether the 3D-printed ECL biosensor can work with multiple samples in different wells without cross-talk. The results show that all wells are working independently of each other, which means that one well reacting does not affect the reaction of any other well. Having multiple wells react independently is crucial for detecting multiple biomarkers simultaneously without cross-talk. The fact that each well was only responsive to its individual analytes gives us confidence that the system would work efficiently in real life.

Some experiments were performed to confirm the reliability of the biosensor. In the first experiment (Fig. 2 (D)), the same glucose concentration was added to both wells to check for consistent ECL response. The ECL signal intensity registered the same value, confirming that the biosensor gives reproducible and reliable results at the same analyte concentration. In the second experiment (Fig. 2 (E)), two different glucose concentrations were put in different wells to see if the biosensor could differentiate between different levels of analytes. Because of this, the ECL signal intensity varied, and thus, we can conclude that the sensor can accurately quantify differences in the analyte concentration. In the last experiment (Fig. 2 (F)), the concentrations of glucose in the wells were exchanged, and the ECL intensity was recorded again. When the positions were changed but the concentrations remained the same, it was found that the intensity was not affected. This indicates that the behaviour of any well was unaffected by the interchange of glucose. The performance results confirm several benefits of the ECL biosensor created from 3DP. The ability to perform several tests simultaneously without interference between the signals is useful for biochemical and diagnostic testing. Additionally, the precision in the quantification of the biomarker supports the strong relationship between the luminescent intensity and analyte concentration, making this an effective and reliable detection method for real-time applications. The system also offers possibilities for high-throughput screening due to its scalability and ability to detect inlets, particularly in the fields of medical diagnostics, biochemistry, and environmental screening. The signal intensity was proportional to the analyte in the well, even when concentrations were switched between wells. This indicates the systematic precision and reliability of the system. The testing also shows that this biosensor can scale for efficient detection that is rapid, low-cost, and accurate in terms of antagonist and through diagnostic testing applications in health care, agricultural, and biological applications.

### Validation of IDE-driven signal amplification

To experimentally validate the contribution of IDEs to ECL signal enhancement, a comparative study was performed using two 3DP biosensor configurations: one incorporating IDEs and the other utilizing planar (non-interdigitated) electrode bands. Both devices were fabricated under identical conditions using conductive PLA filament, and the only difference was the electrode architecture. Figure 2 (G) shows that the IDE-based sensor produced a significantly higher ECL intensity than the non-IDE configuration. Quantitatively, the IDE design resulted in approximately a 2.5-fold increase in luminescence signal, confirming the role of IDEs in facilitating redox cycling and enhancing electron transfer efficiency. The narrow inter-finger gap and alternating polarity of the IDE arms promote continuous oxidation-reduction reactions between adjacent electrode bands, thereby amplifying the luminol–H₂O₂ chemiluminescent response. Photographs of the actual sensor prototypes are also included in Fig. 2 (G) to illustrate the structural differences. The IDE configuration exhibits a more complex electrode pattern that maximizes reactive surface area within a confined footprint. This translates into superior electrochemical performance without increasing device size or fabrication cost. This comparative experiment confirms the mechanical advantage of the IDE layout and demonstrates that the enhanced ECL signal is attributable solely to the electrode design. These findings strongly support our design strategy and reinforce the role of IDEs as a critical feature for achieving high-sensitivity and interference-free multi-analyte detection in the proposed biosensing platform.

### Enzyme optimization

Finally, the sensitivity and reliability of such biosensors largely depend on the enzyme’s ability to catalyze specific biochemical reactions that generate detectable signals. Optimizing the concentration of enzymes ensures that there is a sufficient amount to fully catalyze the target substrate without excess, which could lead to unnecessary costs, potential background noise, or saturation effects that compromise analytical performance. Moreover, it helps to identify the optimal balance where maximum signal output is achieved with minimal reagent consumption, thereby enhancing both the efficiency and cost-effectiveness of the biosensor.

In this study, Figs. 2 (H) and (I), the ECL response is evaluated across a range of LOx and GOx concentrations, respectively. For LOx, the signal increases up to 50 U/mL before plateauing, suggesting that higher enzyme concentrations do not enhance the response further, as the system reaches substrate saturation. A similar pattern was observed for GOx, with optimal ECL output achieved at 10 mg/mL. These findings confirm the most effective enzyme concentrations for achieving high signal intensity without reagent waste, ultimately supporting robust, sensitive, and scalable biosensor design.

Finally, the established detection principle and specificity analysis were carried out under the operating conditions. First, a dual-blank control, both well prepared with the complete assay mixture but without the target analyte, yielded only background-level ECL, confirming that luminol and the assay matrix do not produce a signal in the absence of substrate. Second, a split-well test, one well spiked with the target and the paired well left blank, produced a clear ECL response exclusively in the spiked well, while the blank well remained at baseline. Together, Figs. 2 (J–K) demonstrate that the observed ECL arises from the intended substrate-specific enzymatic reaction and not from non-specific or inter-well effects, thereby reinforcing the optimization and specificity of the assay.

### Analytical performance of ECL device

The analytical performance of the ECL biosensor for the detection of glucose and lactate was evaluated with respect to sensitivity, linear range, and detection limits. The main aim was to see how well the biosensor responds to different concentrations of these two analytes and its effectiveness at multi-analyte detection in real-time. Glucose and lactate were measured in one go, highlighting that the ECL biosensor can be multiplexed. Blood lactate is routinely interpreted in the 0.5–2.2 mM physiological window, with hyperlactatemia > 2 mM and severe lactic acidosis typically > 4 mM; values ≥ 2 mM are already clinically actionable in sepsis, ischemia, trauma, perioperative monitoring, neonatal care, and sports physiology.

**Fig. 3:**
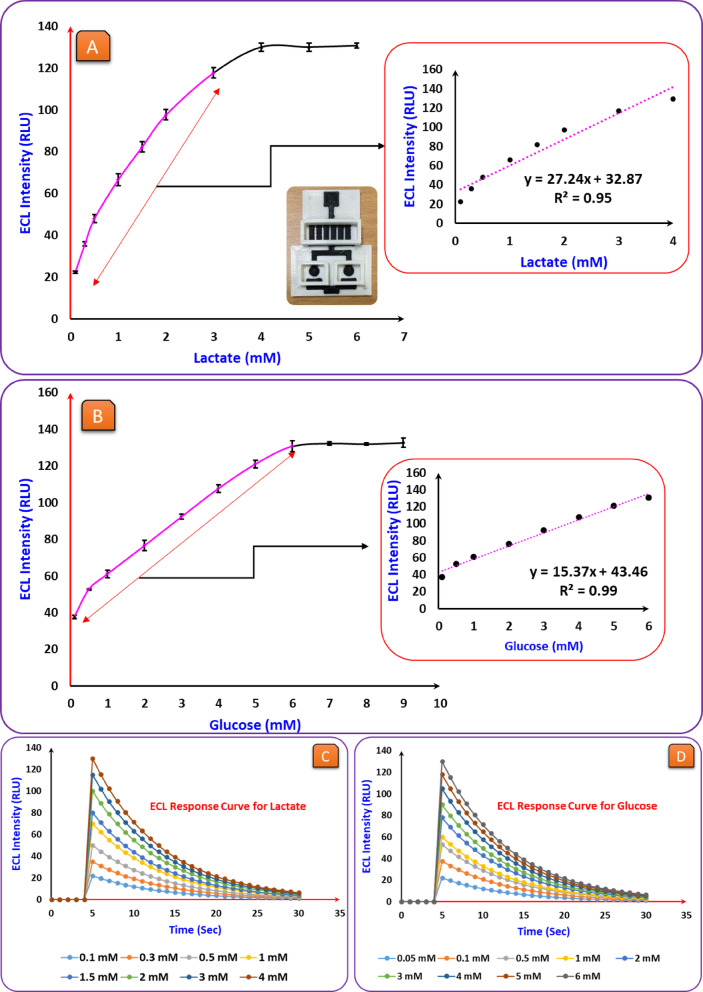
(**A**) Calibration curve for lactate detection showing the relationship between ECL intensity and lactate concentration: Lactate (1 µM to 6 mM), voltage (7 V), Luminol (4 mM), pH (9.5), LOx (50 UN/mL). (**B**) Calibration curve for glucose detection, illustrating the increase in ECL intensity with increasing glucose concentration: glucose (1 µM to 9 mM), voltage (7 V), Luminol (4 mM), pH (9.5), GOx (10 mg/mL). Each experiment was conducted three times (*n* = 3). (**C**) and (**D**) ECL response curves of the fabricated 3DP biosensor for lactate and glucose detection at various concentrations.

Consequently, our Limit of detection (LOD) of 80 µM (0.08 mM) is and (linear range between 0.1 mM and 4 mM) well lower than the lower bound of the normal physiological range, enabling (i) early detection of subtle metabolic shifts, (ii) confident measurements after high dilutions of real samples, and (iii) robust operation in matrices with variable background. The LOD = (3 * δ)/ m, where δ = standard deviation and m = slope of the corresponding calibration curve^49^.

As observed in Fig. 3 (A), the ECL intensity rose proportionally with lactate concentration up to 4 mM, after which it saturated due to enzyme kinetics and electrode surface limitations. The inset graph further confirms a strong linear correlation (R² = 0.9453), emphasizing the sensor’s reliability in lactate quantification. While the full lactate concentration range tested extended up to 7 mM, the calibration curve was modeled using a linear regression within the 0.1 mM to 4.0 mM range, which is most relevant for physiological and clinical applications. This region exhibited a strong linear relationship between ECL intensity and lactate concentration (R² = 0.9453), supporting the use of a linear fit. At concentrations beyond 4.0 mM, a slight deviation from linearity was observed, which can be attributed to enzyme saturation kinetics and surface site saturation at the electrode. Such saturation effects are typical in enzymatic biosensing platforms, where at higher substrate concentrations, the enzyme active sites become fully occupied, and additional analyte does not proportionally increase signal output.

Furthermore, the high surface activity of the IDE-based ECL biosensor helps sustain linearity over a broader dynamic range, but beyond a certain threshold, the redox cycling efficiency and luminol-H_₂_O_₂_ reaction kinetics approach their upper limits, resulting in signal plateauing. The linear model was applied only within the range demonstrating first-order kinetics to preserve analytical accuracy and ensure consistent quantification. This approach is consistent with previous studies on ECL biosensing platforms, where linear dynamic ranges are reported up to enzyme saturation points.

To evaluate the biosensor’s response to glucose (Fig. 3. B), the concentration was systematically varied from 1 µM to 10 mM, covering a broad physiological range. The obtained linear range extended from 0.1 mM to 5 mM, indicating the sensor’s ability to measure glucose levels within biologically relevant concentrations accurately. The LOD was determined to be 0.1 mM, confirming the sensor’s high sensitivity. Figure B shows that the ECL intensity increased linearly with glucose concentration up to 5 mM, beyond which the signal began to plateau due to the saturation of available reactive sites. The concentration was systematically varied from 1 µM to 10 mM to evaluate the biosensor’s response to glucose, covering a broad physiological range. The obtained linear range extended from 0.1 mM to 5 mM, indicating the sensor’s ability to accurately measure glucose levels within biologically relevant concentrations. The LOD was determined to be 0.1 mM, confirming the sensor’s high sensitivity. Figure B shows that the ECL intensity increased linearly with glucose concentration up to 5 mM, beyond which the signal began to plateau due to the saturation of available reactive sites.

Finally, to assess the real-time ECL performance of the 3DP biosensor, time-resolved ECL intensity curves were recorded for lactate and glucose at different concentrations. In Figs. 3(C) and (D), the ECL signal is seen to remain at or near baseline for the first 5 s, followed by a sharp increase in intensity due to rapid enzyme catalysis and electron transfer events. This is immediately succeeded by a gradual exponential decay, likely arising from luminol oxidation and substrate exhaustion. Higher analyte concentrations yielded stronger peak signals, establishing a clear correlation between ECL intensity and analyte level. This behaviour confirms the biosensor’s reliability for simultaneous, concentration-dependent detection of lactate and glucose.

### Reliability, reproducibility, and specificity of the ECL biosensor

Blank control experiments were performed to evaluate the background signal and confirm the specificity of the ECL response. These blank samples contained the same components of the assay, including electrolyte, buffer solution, and luminol, but did not contain the target analytes (glucose and lactate) or the respective enzymes (GOx and LOx). Under these conditions, no measurable ECL signal was observed, confirming the absence of non-specific luminescence in the system. This indicates that the ECL signal is generated only through enzyme-catalyzed reactions involving the target analytes, validating the selectivity of the biosensor. In addition, the lack of a baseline signal defines a clear limit of blank and supports the previously identified high signal-to-noise ratio needed to achieve sensitive and reliable detection. These findings confirm that the biosensor does not give false-positive responses and is capable of accurate quantification in real-world diagnostic assays.

**Fig. 4:**
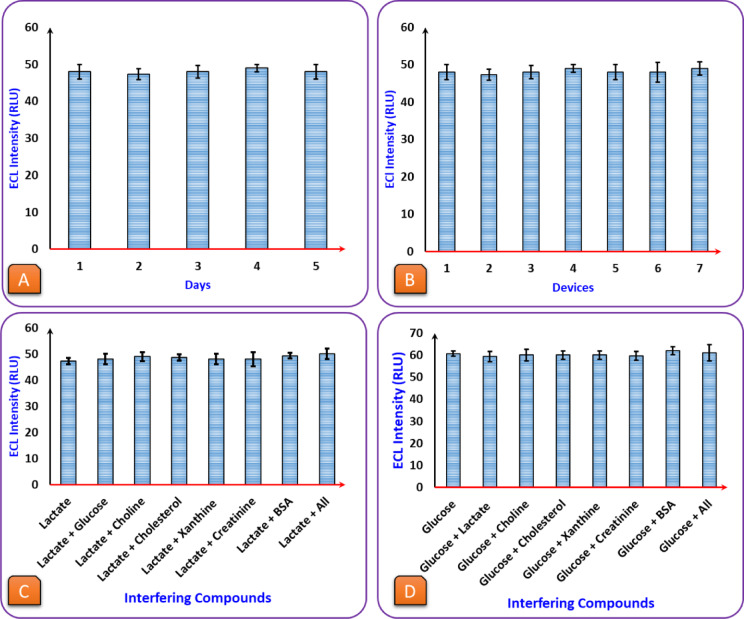
(**A**) Stability assessment of the ECL biosensor over five consecutive days: Lactate (0.5 mM), voltage (7 V), Luminol (4 mM), pH (9.5), LOx (50 UN/mL). (**B**) Reproducibility evaluation across seven independently fabricated biosensors: Lactate (0.5 mM), voltage (7 V), Luminol (4 mM), pH (9.5), LOx (50 UN/mL). (**C**) Selectivity study testing the effect of potential interfering compounds, including glucose (10mM), choline (1 mM), cholesterol (10 mM), xanthine (1mM), BSA (40 g/L), and creatinine (1 mM), on lactate detection. (**D**) Selectivity study testing the effect of potential interfering compounds, including glucose (1 mM), choline (1 mM), cholesterol (1 mM), xanthine (1 mM), Lactate (10 mM), BSA (40 g/L), and creatinine (1 mM), on Glucose detection.

Then, the ECL biosensor was assessed for stability over time, reproducibility across devices, and the ability to detect target analytes within complicated samples. These characteristics contribute to the reliability of the biosensor for application in biomedical and diagnostic settings in real-world situations. Additionally, the repeatability of the results confirms that the biosensor exhibits adequate performance and is capable of real-time monitoring and point-of-care testing for detecting a target metabolite. To check stability over time, the biosensor’s ECL intensity was measured over multiple days (Fig. 4. A). The signal moves uniformly and unchanged, which means the biosensor maintains its timely and accurate response. It shows that important parts like the electrodes, enzyme action, and ECL reaction with luminol are still working well and not breaking down. This stability test confirms the biosensor can be used for continuous monitoring, making it suitable for diagnostic applications. Subsequently, several biosensor devices made in the same way were assessed to check their reproducibility (Fig. 4. B). Each device produced nearly identical ECL signals; thus, the fabrication process is reproducible and reliable. This ensures that every sensor works the same way, which is essential for mass production. Having a reproducible system makes this biosensor a strong candidate for widespread use in diagnostic labs and point-of-care testing.

To evaluate the selectivity of the biosensor in complex biological matrices, we initially tested common serum-based small-molecule interferents such as glucose, choline, cholesterol, xanthine, and creatinine, all of which are electrochemically active and capable of interfering with enzymatic or luminol-based systems (Fig. 4. C and D). However, since serum also contains high levels of proteins, we conducted additional interference testing using BSA, which is within the physiological range for human serum albumin. The presence of BSA did not produce any noticeable change in ECL intensity when compared to protein-free controls, indicating that the biosensor surface is sufficiently resistant to non-specific protein binding and matrix fouling. This confirms the selectivity of the system not only toward target analytes but also in the presence of protein-rich biological fluids. The outcome verified that the ECL intensity of the biosensor remains unaffected, which confirmed its capability to detect glucose and lactate in the presence of other substances. ECL-based biosensors exhibit remarkable stability, reproducibility, and selectivity, rendering them highly attractive electrochemical-based analytical techniques for widespread diagnostics and bio-analysis. Their performance is resilient over time; they enable results that correlate with devices, and other molecules minimally influence them in the presence of the desired biomarker. Because of these characteristics, ECL biosensors can be used for real-time monitoring, point-of-care diagnosis, and possible implementation into clinical environments in the future. It can provide a quick, scalable, and highly reproducible solution for healthcare and research.

### Real sample analysis using ECL biosensor

Real-sample analysis is essential to demonstrate practical applicability, resilience to matrix effects, and agreement with clinical assays. This work analyzed de-identified human serum from Metro Lab Diagnostic Center (Nagpur, India) exactly as in the calibration workflow, with the same optimized bias, luminol/pH, enzyme loading, smartphone imaging, and ROI-based background subtraction. The results (Table 1) show close agreement with the clinical method, glucose: 4.55 vs. 4.70 mM (96.80% accuracy); lactate: 1.27 vs. 1.30 mM (97.69% accuracy), indicating minimal matrix interference. These outcomes substantiate the clinical relevance and reliability of the IDE-ECL platform in serum and support its suitability for near-patient deployment.

**Table 1:** Real sample analysis.

Analyte	Clinical method (mM)	Using Proposed Biosensors (mM)	Obtained ECL Intensity (RLU)	Accuracy = (Proposed /Clinical)*100
Glucose	4.7	4.55	113.39	96.80
Lactate	1.3	1.27	67.46	97.69

Table 2 shows the comparative analysis of ECL biosensing technologies based on fabrication methods, detection limits, and linear range.

**Table 2:** Comparative analysis of ECL biosensing Technologies.

Biosensor Type	Fabrication Method	Target Analytes	Detection Limit	Linear Range	Estimated Cost per Device	Simultaneous Detection	References
Paper-based BPE-ECL	Screen-printing of carbon ink on paper	H_2_O_2_, Glucose	1.75 µM (H_2_O_2_), 0.017 mM (glucose)	5–5000 µM (H_2_O_2_)	$0.015	Not Possible	^16^
Open BPE-ECL on paper	Wax-screen-printing for microfluidic channels	TPA, H_2_O_2_	8.70 µM (TPA), 46.6 µM (H_2_O_2_)	10–1000 µM (TPA), 50–5000 µM (H_2_O_2_)	–	Not Possible	^43^
Cloth-based BPE-ECL	Wax and carbon ink screen-printing on cloth	Glucose, H_2_O_2_	0.195 mM (glucose), 0.024 mM (H_2_O_2_)	-	$0.015	Not Possible	^22^
Smartphone-based 3D-printed ECL	3D-printing with conductive PLA electrodes	Glucose	60 µM	Up to 5 mM	–	Not Possible	^50^
Single-Electrode ECL System	Resistance-induced potential difference	Multiplex ECL analysis	H_2_O_2_	5.0 to 100.0 µM	–	Not Possible	^48^
Bipolar Electrode Arrays	Microfluidic bipolar system	Chemical imaging, multiplex sensing	H_2_O_2_	-	–	Possible	^26^
Microfluidic Bipolar ECL	Dual-channel bipolar system	TPrA, H_2_O_2_	TPrA- 0.1µM, H_2_O_2_– 2.5 µM,	TPRA- 0.1 µM to 1 mM, H_2_O_2_- 5 µM to 0.1 mM	–	Not Possible	^51^
Electrochemical ECL Device (Three-Electrode Device)	Glassy Carbon Electrode	Lactate	2.52 µM	5–30 µM	–	Not Possible	^52^
Colorimetric Sensing	Paper-Based Device	Lactate	100 µM	1 mM to 10 mM	–	Not Possible	^53^
3D Printed	3D-printed ECL sensor with interdigitated electrodes (IDEs)	Glucose, Lactate	0.1 mM (glucose), 80 µM (lactate)	0.1–5 mM (glucose), 0.1–4 mM (lactate)	~ $0.87	Possible	This work

## Conclusion

This work presents a portable, low-cost 3D-printed ECL platform based on IDEs that enables simultaneous detection of glucose and lactate on a single device. The architecture delivers strong signal quality with negligible cross-talk and good reproducibility across physiologically relevant ranges. The biosensor exhibits linear responses from 0.1 to 5 mM for glucose (LOD: 0.1 mM) and 0.1–4 mM for lactate (LOD: 80 µM), including validation with human blood serum, underscoring suitability for near-patient use. The device demonstrates a scalable and cost-effective route toward practical metabolic monitoring. In the current study, our primary focus is on device novelty, the simultaneous multi-analyte detection, and system portability/cost. The smartphone is integral as a compact, accessible imaging and power interface within a light-shielded 3D-printed cradle, while open-source ImageJ was used for intensity extraction to ensure transparent and widely comparable quantification as we established device performance. Building on this foundation, our ongoing follow-up work targets full automation: a phone-native image pipeline (exposure lock, ROI/background handling, temporal integration) coupled with deep-learning models for on-device concentration prediction and true end-to-end, PC-free operation. This roadmap advances the present prototype toward a fully automated, field-deployable metabolic monitoring system.

## Data Availability

No datasets were generated or analysed during the current study.
